# A Knotless Technique for Kidney Transplantation in the Mouse

**DOI:** 10.1155/2012/127215

**Published:** 2012-07-17

**Authors:** Song Rong, Alfor G. Lewis, Uta Kunter, Hermann Haller, Faikah Gueler

**Affiliations:** ^1^Division of Nephrology, Hannover Medical School, 30625 Hannover, Germany; ^2^The Transplantation Center, Affiliated Hospital, Zunyi Medical College, Zunyi 563003, China; ^3^Division of Developmental Biology, Cincinnati Children's Hospital Research Foundation, Cincinnati, OH 45229, USA; ^4^Division of Nephrology and Immunology, RWTH University of Aachen, 52062 Aachen, Germany; ^5^Phenos GmbH, 30625 Hannover, Germany

## Abstract

Mouse models of kidney transplantation are important to study molecular mechanisms of organ transplant rejection as well as to develop new therapeutic strategies aimed at improving allograft survival. However, the surgical technique necessary to result in a viable allograft has traditionally proven to be complex and very demanding. Here, we introduce a new, simple, and rapid knotless technique for vessel anastomosis wherein the last stitch of the anastomosis is not tied to the short end of the upper tie as in the classical approach but is left free. This is a critical difference in that it allows the size of the anastomosis to be increased or decreased after graft reperfusion in order to avoid stenosis or bleeding, respectively. We compared the outcome of this new knotless technique (*n* = 175) with the classical approach (*n* = 122) in terms of local thrombosis or bleeding, time for anastomosis, and survival rates. By this modification of the suture technique, local thrombosis was significantly reduced (1.1% versus 6.6%), anastomosis time was less, and highly reproducible kidney graft survival was achieved (95% versus 84% with the classical approach). We believe that this knotless technique is easy to learn and will improve the success rates in the technically demanding model of mouse kidney transplantation.

## 1. Introduction

Mouse models are essential in the study of mechanisms of acute [1–3] and chronic kidney transplant rejection [4–6]. They are especially important for the investigation of the role of certain genes in transplant rejection by studying knockout, transgenic, congenic, and inbred mouse strains [7]. Secondly, new experimental therapeutic strategies can be evaluated which only require low amounts of compound, which are often cost and time intensive in their production. Furthermore, a multitude of antibodies and molecular approaches for mouse research is available.

Mouse kidney transplant models have been described in the literature with varying mortality rates due to technical problems [8, 9]. Complications of kidney transplantations in mouse and rat are similar and are frequently related to the vessel anastomosis; thrombosis, hemorrhage, thrombo-embolism, ischemia-reperfusion damage, and stenosis have been reported [10]. In this report, we describe the results of 297 mouse kidney transplantations where we compared the standard technique (*n* = 122) with technical modifications (*n* = 175), especially of the vessel anastomosis. In the classical approach, the last suture of the vessel wall is tied to the short tail of the stay suture. This technique does not allow modification of the size of the anastomosis after reperfusion. In the new knotless technique, the last stitch is not tied but left free, which allows the surgeon to increase or decrease the size of the anastomosis if necessary. Following kidney transplantation outcomes of both techniques (the knotless and the classical technique) were compared in terms of operative time and surgery-associated complications such as thrombosis and bleeding. In addition, long-term survival and renal function with the new knotless technique was monitored over 12 weeks. 

## 2. Materials and Methods

### 2.1. Animals

Twelve- to sixteen-week-old (18–25 g body weight) C57Bl/6(H2b), 129/Sv(H2b), and BALB/c(H2d) mice (Charles River Sulzfeld, Germany) were studied since these are the strains most frequently used for transplant studies. Animals were cared for in accordance with our institutional guidelines for experimental animals, and all experiments were approved by the local animal protection committee. 

### 2.2. Experimental Design

Both techniques (the classical and the knotless) were utilized in allogenic and isogenic transplantations. With the classical technique 66 allogenic and 56 isogenic kidney transplantations (total *n* = 122) were performed. With the new knotless technique, 106 allogenic and 69 isogenic kidney transplantations were performed (total *n* = 175). Survival was monitored for 4 days and if a recipient died prematurely, an autopsy was performed. In addition, 15 long-term experiments were performed for 12 weeks with monitoring of weight gain and renal function.

### 2.3. Surgical Technique

#### 2.3.1. Donor Operation and Kidney Perfusion

Mice were anesthetized via inhalation with isoflurane (2% in air; flow 200 mL/min). Following a midline abdominal incision from sternum to pubis the left kidney, aorta and inferior vena cava (IVC) were fully exposed. The small vessels (including the left adrenal vessels), the left lumbar vein, and the underlying vascular branches running to the lumbar vessels were carefully cauterized and cut. The left ureter was dissected free of surrounding tissue from the renal hilum to the bladder and cut near the level of the bladder. The aortic region between the left and right renal arteries (approximately 2 mm in length) was mobilized. The infrarenal IVC and aorta were separated, and curved forceps were passed beneath the aorta at this point to place a loose tie of 7-0 silk suture around this vessel. The aorta below the right renal artery, distal aorta, and IVC were successively cross-clamped with two 5 mm microvascular Yasargil clamps, and the left renal vein was transected at the vena cava. The aorta was perfused with 1 mL of normal saline solution (NS). The ligature was then tightened, and the aorta was cut below the ligature and below the proximal clamp. The kidney and associated vessels were completely freed by cauterizing all tissue surrounding the major vessels, and then removed and stored in NS at 4°C.

### 2.4. Recipient Operation

**Figure 1:**
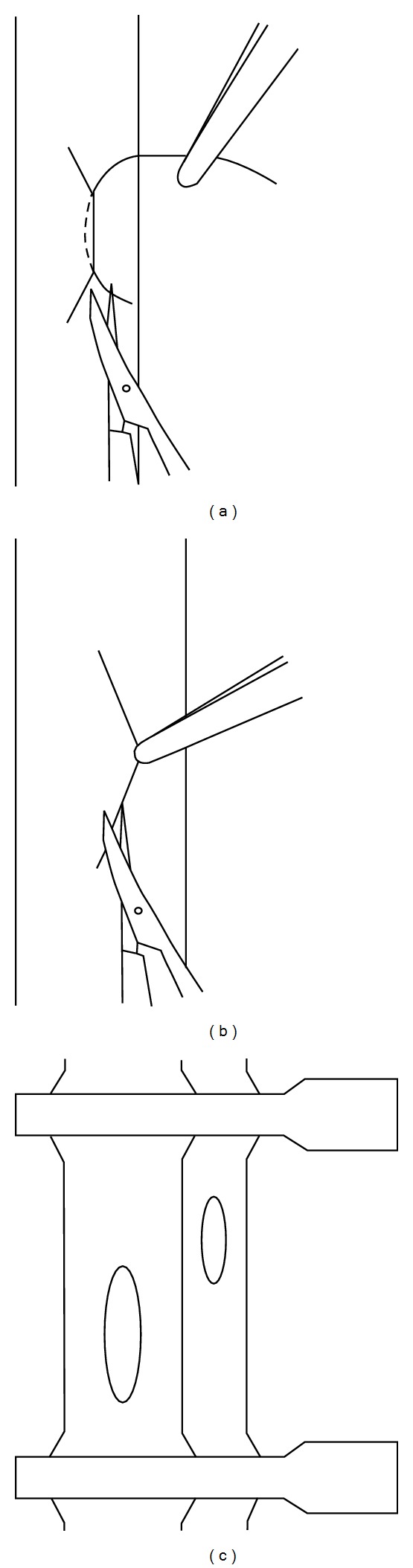
Preparation of the anastomosis. Lifting of the vessel wall by a single suture for incision of the aorta (a) and incision of the vena cava (b). Incision of the inferior vena cava is slightly below that of its aortic counterpart (c).

The abdomen was opened via midline incision, and the bowel was moved to the right abdomen and covered with moist gauze. Following, removal of the left kidney, the infrarenal aorta and IVC were carefully isolated, and every large branch was cauterized in each. A section of aorta and IVC (approximately 4 mm in length) was dissected, and then cross-clamped with two microvascular clamps, proximally and distally. A 10/0 Prolene suture needle was placed through a full thickness of the aorta in a proximal-distal manner, and an elliptical arteriotomy of approximately 1 mm was achieved by gentle upward traction on the suture while cutting directly below (and in contact with) the lower face of the needle with fine, curved scissors (Figure 1(a)). The IVC was cut longitudinally with sufficient length (approximately 1.5 mm) to permit anastomosis without risking renal vein stenosis (Figure 1(b)); this incision was slightly below its aortic counterpart (Figure 1(c)). Both vessels were then thoroughly irrigated with NS to flush out any blood clots.

### 2.5. Implantation of the Kidney Graft

**Figure 2:**
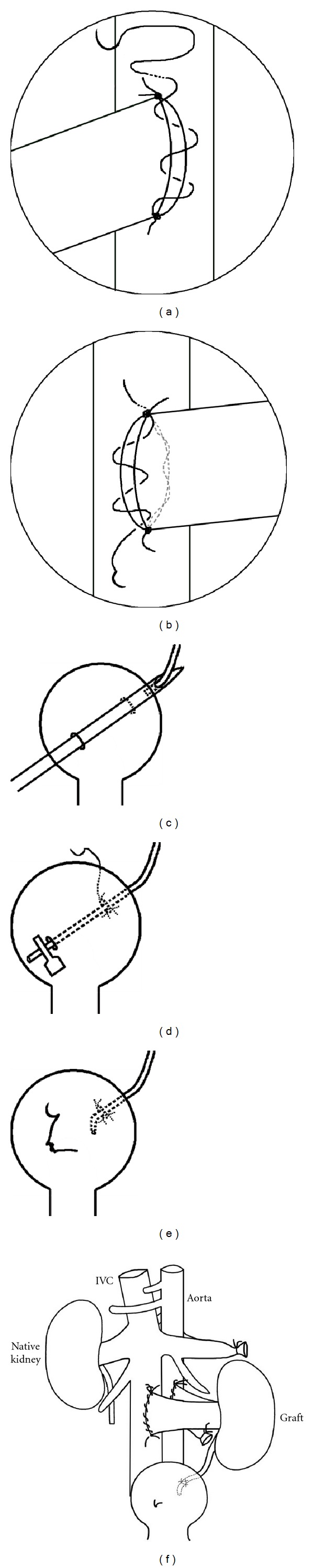
Arterial anastomosis ((a) and (b)) was performed in an end-to-side manner between the donor renal artery and recipient aorta. For ureteral anastomosis ((c)–(e)), the free end of the ureter was stripped of fat and introduced into the needle lumen with fine forceps, and the needle was gently withdrawn from the bladder, with the ureter accompanying it (c). Once it had exited the lower right bladder wall puncture site, the free end of the ureter was immediately clipped with a microvascular clamp, to avoid its retraction into the bladder (d). The ureter was anastomosed and the puncture wound in the bladder repaired (e). Situation after transplantation showing position of the transplanted kidney relative to the nephrectomy site and the anastomoses of the major blood vessels (f).

The arterial anastomosis (Figures 2(a) and 2(b)) was performed in an end-to-side manner between the donor and recipient aorta. The proximal and distal ends of the anastomosis were first sutured using two separate 10/0 Prolene sutures (one at either pole). After tying, the two long sutures with the needle were left in place. The left wall of the anastomosis was sewn continuously with two evenly spaced stitches in a distal-proximal direction. After the last stitch, the suture was passed through a partial thickness of vessel wall, above the upper stay suture tie, and simultaneous gentle traction was applied to the short end of the lower suture tie. Importantly, in this new knotless technique, the last stitch was not tied to the short end of the upper tie. The graft kidney was turned over to its normal position, and the right wall was then continuously sewn in a proximal-distal manner with three stitches (the final suture emerged near the distal tie). This was not tied to the short end of the lower suture but was cut to leave a free length of about 2-3 mm. The venous anastomosis was performed with the same suturing procedure, but four or five stitches were needed for each side of the anastomosis (again, the final stitch was left as a free end of similar length to that described above). After completing both anastomoses, gentle pressure was applied to the sutured area via a dry swab stick for 10–20 seconds, and both clamps were removed. 

As a separate comparison group (*n* = 122), anastomoses were also performed using the classical approach whereby after completing the left vessel wall anastomosis, the suture was tied to the short tail of the upper stay suture, and the needle was removed. The right vessel wall anastomosis was then completed in a continuous manner using the long tail (with needle) from the upper stay suture, and, upon reaching the lower stay suture, it was tied to the short tail of the latter and then cut.

### 2.6. Ureteral Implantation

Ureteral implantation was generally performed as described previously [11] with a slight, but important modification. Instead of drawing the ureter through the bladder with curved forceps, the free end of the ureter was stripped of fat and introduced into the needle lumen, and the needle was gently withdrawn from the bladder, with the ureter accompanying it (Figure 2(c)). Once it had exited the lower right bladder wall puncture site, the free end of the ureter was immediately clipped with a microvascular clamp, to avoid its retraction into the bladder (Figure 2(d)). Periureteral fat tissue was fixed to the exterior wall of the bladder dome using a 10/0 Ethilon suture (two or three interrupted sutures). The ureteral region that had previously been stripped free of fat was incised so that only a short, undamaged length retracted into the bladder. This site was closed via a “figure of eight” stitch using a 10/0 Ethilon suture (Figure 2(e)). The position of the transplanted kidney relative to the nephrectomy site and the anastomoses of the major blood vessels is shown in Figure 2(f).

Finally, the abdominal cavity was closed with 4/0 silk suture. After closure of body wall and skin, 1.5 mL NS was administered subcutaneously, and the mouse was placed on an electric warming blanket until fully awake. The mouse was then returned to its regular housing with free access to food and water. 

### 2.7. Contralateral Nephrectomy

The right kidney was removed immediately at the time of transplantation or, in the long-term survival experiments, 4 days after the transplantation.

### 2.8. Renal Function

Transplanted mice were studied for renal function and survival. Serum creatinine levels were measured by an automated method (Beckman Analyzer, Germany) [12].

### 2.9. Outcome Parameters

Times for arterial and venous anastomosis were assessed. Thrombosis and local bleeding as reasons for mortality were assessed intraoperatively and also by autopsy within the first 4 days after transplantation. 

### 2.10. Statistical Analysis

Data are presented as mean ± the standard error of the mean (SEM). Differences were considered as significant at *P* < 0.05. A *t*-test was used to test the mean differences in times for arterial and venous anastomoses between old and new knotless technique groups. The Chi-square test was used to assess differences in complication rates between two groups. 

## 3. Results

### 3.1. Operative Time and Complication Rate

Operative time and complication rates were compared between the classical technique (*n* = 122) and the new modified technique (*n* = 175). In both groups allogenic and isogenic transplantations were performed. Total operative time was significantly reduced with the new knotless technique, as evidenced by the reduced times required to prepare the arterial and venous anastomoses (Table 1). In addition, a significantly reduced incidence of complications (Table 2) such as thrombosis (1.1% versus 6.6% with the classical technique; *P* < 0.05) and improved survival was achieved with the new knotless technique. The technical success rate was defined as survival more than 4 days following the removal of the native contralateral kidney (life supporting model). The survival improved significantly from 84.4% with the classical technique to 95.4% with the new modified knotless technique (*P* < 0.005). During the first four days, no urological complications (such as visible hydronephrosis due to stenosis at the site of ureteral anastomosis) were observed.

### 3.2. Long-Term Survival, Renal Function, and Morphology

**Figure 3:**
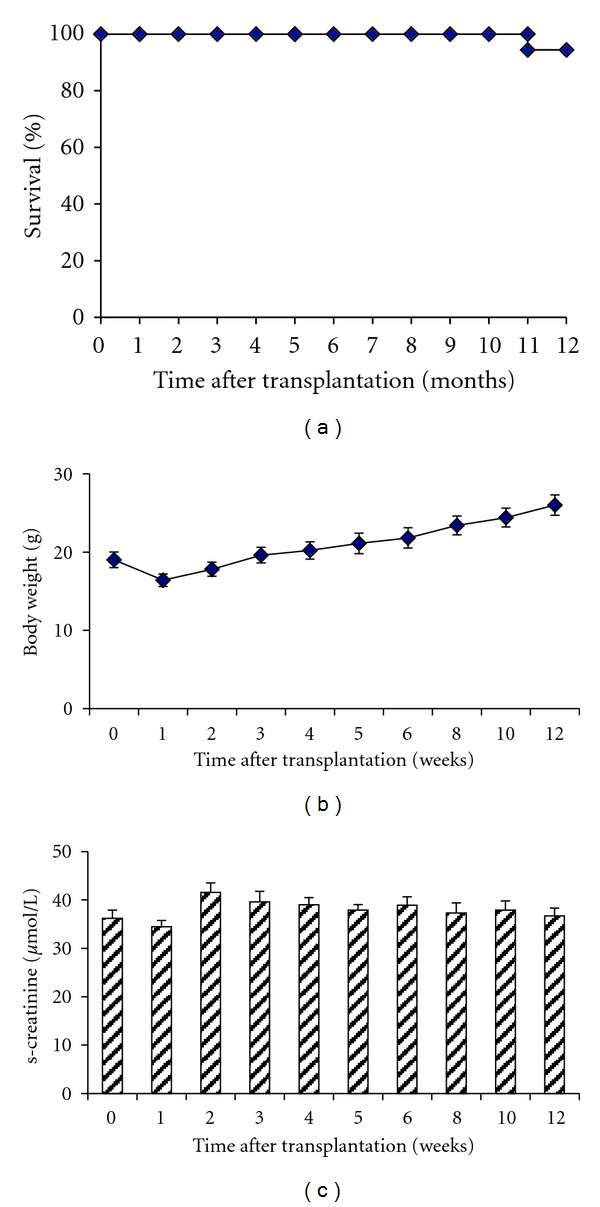
Isogenic transplantation was performed in 15 mice, and survival was monitored over 12 weeks. Only one kidney transplant recipient died during the observation period, resulting in 93% long-term survival (a). Recipients showed constant weight gain, indicating normal growth (b). Renal function remained stable over the observation period of 12 weeks (c).

In addition to short-term outcome, renal function and long-term survival were studied in 15 isogenic kidney transplant recipients. Long-term survival over 12 weeks with the new knotless technique was 95%, and the mice constantly gained body weight indicating normal growth and well-being (Figures 3(a) and 3(b)). Serum creatinine increased slightly but not significantly after transplantation and remained stable during the observation period of 12 weeks (Figure 3(c)). 

## 4. Discussion

Murine kidney transplantation has gained widespread use because of the generation of a large number of genetically modified mice and the availability of a broad variety of molecular probes and techniques that enable subsequent analysis. However, technical failure during surgery, most commonly presenting as bleeding or stenosis at the sites of vessel anastomosis are still major obstacles to long-term success after mouse kidney transplantation. 

We developed a new modified technique to simplify the procedure. In contrast to heterotopic heart transplantation [13], the kidney transplant model offers an opportunity to study a life-supporting model following bilateral nephrectomy of the native kidneys of the recipient. 

As described by Martins 2006 [9] kidney transplantation is challenging, and the success rates of even experienced surgeons vary between 40 and 70% [4, 14]. The highest survival rates in the literature have been reported by Zhang and coworkers ranging between 79 and 90% (in the last 50 operations) [8]. 

The success of kidney transplantation in small animals depends upon general complications, that is, hypothermia, hypovolemia, intraoperative blood loss, or infections and these are surgeryrelated [10]. Anastomotic bleeding, stenosis, and thrombosis are severe complications causing subsequent allograft failure due to technical problems rather than real rejection episodes. Therefore, the establishment of high-quality anastomoses plays a key role in achieving good allograft survival and highly reproducible experimental results. In a detailed study by Martins [9] arterial thrombosis was described in 46% of transplantations due to nonmatching aortotomy-aorta patch sizes, major bleeding, or excessive aorta manipulation during anastomosis. The new knotless suture technique offers a rapid and easily performed technique which allows the tightness of the anastomosis to be altered after graft reperfusion, if necessary. Conversely, if arterial or venous stenosis is encountered, the anastomosis can be enlarged by gently drawing the free tails of the proximal and distal anchor stitches of the appropriate vessel in opposite directions, thereby increasing the diameter of the suture loop so that flow can be restored without leakage. Consequently, our modified suturing procedure can effectively eliminate the two major complications associated with this animal model of renal transplantation: anastomotic bleeding and stenosis/thrombosis. With the knotless technique, the incidence of thrombosis was significantly reduced compared to the classical technique. In addition to reducing the rate of thrombosis, the operative time for the arterial and venous anastomosis was significantly reduced by the new knotless technique. The length of the warm ischemic time contributes to (and is a major risk factor for) acute renal failure after transplantation [15], especially when bilateral nephrectomy is planned at the time of transplantation. In this situation the incidence of acute renal failure due to prolonged warm ischemia might contribute to higher mortality rates. Another complication of kidney transplantation is caused by injury to the periureteral tissue resulting in ureteral ischemia and subsequent ureteral necrosis and leakage. Instead of retracting the ureter through the bladder with forceps [11], we gently drew it through the bladder wall by placing it into a needle lumen. We believe that this might reduce local tissue damage to the ureter and helps to avoid urinary necrosis and leakage [10]. We did not observe hydronephrosis or visible swelling of the kidney due to gross infectious nephritis during the first four days when autopsies were performed. However, we cannot estimate the rate of possible late complications (due to ureteral stenosis and subsequent hydronephrosis from scarring at the anastomosic site) at later time points. The mice in the survival groups did not show general signs of severe infection, which indicates the absence of pyelonephritis or inflammation of the kidney. In summary, we have shown that by modifying the technique for arterial and venous anastomosis that the operative time was reduced and by providing a means to modify the size of the anastomosis, the incidence of local thrombosis can also be reduced significantly. The new technique resulted in improved survival rates and should increase the number of studies utilizing the mouse as a model of kidney transplantation.

## Figures and Tables

**Table 1:** Operative times in minutes.

	Old technique (*n* = 122)	New knotless technique (*n* = 175)
Time for arterial anastomosis (min)	9.2 ± 0.09	7.5 ± 0.06^∗^
Time for venous anastomosis (min)	9.1 ± 0.1	7.5 ± 0.05^∗^

*P* < 0.005.

**Table 2:** Complication rates with old versus new knotless technique.

	Old technique (*n* = 122)	New knotless technique (*n* = 175)
	Case (%)	Case (%)
Thrombosis	8 (6.6)	2 (1.1)^∗^
Local bleeding	4 (3.3)	1 (0.6)
Success rate	103 (84.4)	167 (95.4)^∗∗^

*P < 0.05, **P < 0.005.

## References

[1] Einecke G, Melk A, Ramassar V (2005). Expression of CTL associated transcripts precedes the development of tubulitis in T-cell mediated kidney graft rejection. *American Journal of Transplantation*.

[2] Famulski KS, Sis B, Billesberger L, Halloran PF (2008). Interferon-*γ* and donor MHC class I control alternative macrophage activation and activin expression in rejecting kidney allografts: a shift in the Th1-Th2 paradigm. *American Journal of Transplantation*.

[3] Gueler F, Rong S, Gwinner W (2008). Complement 5a receptor inhibition improves renal allograft survival. *Journal of the American Society of Nephrology*.

[4] Mannon RB, Kopp JB, Ruiz P (1999). Chronic rejection of mouse kidney allografts. *Kidney International*.

[5] Cheng O, Thuillier R, Sampson E (2006). Connective tissue growth factor is a biomarker and mediator of kidney allograft fibrosis. *American Journal of Transplantation*.

[6] Franceschini N, Cheng O, Zhang X, Ruiz P, Mannon RB (2003). Inhibition of prolyl-4-hydroxylase ameliorates chronic rejection of mouse kidney allografts. *American Journal of Transplantation*.

[7] Gueler F, Rong S, Mengel M (2008). Renal urokinase-type plasminogen activator (uPA) receptor but not uPA deficiency strongly attenuates ischemia reperfusion injury and acute kidney allograft rejection. *Journal of Immunology*.

[8] Zhang Z, Schlachta C, Duff J, Stiller C, Grant D, Zhong R (1995). Improved techniques for kidney transplantation in mice. *Microsurgery*.

[9] Martins PN (2006). Learning curve, surgical results and operative complications for kidney transplantation in mice. *Microsurgery*.

[10] Pahlavan PS, Mehrabi A, Kashfi A (2005). Guidelines for prevention and management of complications following kidney transplantation in rats. *Transplantation Proceedings*.

[11] Han W-R, Murray-Segal LJ, Mottram PL (1999). Modified technique for kidney transplantation in mice. *Microsurgery*.

[12] Gueler F, Rong S, Park JK (2002). Postischemic acute renal failure is reduced by short-term statin treatment in a rat model. *Journal of the American Society of Nephrology*.

[13] Hasegawa T, Visovatti SH, Hyman MC, Hayasaki T, Pinsky DJ (2007). Heterotopic vascularized murine cardiac transplantation to study graft arteriopathy. *Nature Protocols*.

[14] Coffman TM, Geier S, Ibrahim S (1993). Improved renal function in mouse kidney allografts lacking MHC class I antigens. *Journal of Immunology*.

[15] Wang JJ, Hockenheimer S, Bickerstaff AA, Hadley GA (2009). Murine renal transplantation procedure. *Journal of Visualized Experiments*.

